# Retroperitoneal cystic schwannoma: A case report with review of literature

**DOI:** 10.4103/0970-9371.73299

**Published:** 2010-10

**Authors:** Aparna Narasimha, ML Harendra Kumar, R Kalyani, M Madan

**Affiliations:** Department of Pathology, Sri Devaraj Urs Academy of Higher Education and Research, Tamaka, Kolar, India; 1 Department of Surgery, Sri Devaraj Urs Academy of Higher Education and Research, Tamaka, Kolar, India

**Keywords:** Retroperitoneal schwannoma, cystic degeneration, benign, fine needle aspiration cytology

## Abstract

The occurrence of retroperitoneal schwannoma is uncommon and its presence may only be expressed by insidious onset of nonspecific symptoms such as vague abdominal pain. Imaging modalities like computed tomography and magnetic resonance imaging may demonstrate the tumor, but due to heterogeneity and degeneration in some tumors, it may mimic malignancy. So, fine needle aspiration cytology followed by tissue sampling through needle biopsies may be essential to confirm the diagnosis prior to the surgery. This case is reported for its rare clinical presentation, having duration of more than 40 years with cystic degenerative changes.

## Introduction

Retroperitoneal schwannomas are benign tumors arising from Schwann cells of the peripheral nerve sheath.[1] Benign schwannomas are generally slow growing and painless, occurring predominantly in females between the second and fifth decades of life.[2] Preoperative diagnosis is very difficult because of the site and lack of specific features on the ultrasonography, computed tomography (CT) or magnetic resonance imaging (MRI).[3] Guided fine needle aspiration cytology (FNAC) of retroperitoneal mass lesions is widely practiced in several institutions where the facilities of standard imaging techniques and cytopathology are available. Cytopathological examination of the material obtained by guided FNAC may offer quick and specific diagnosis and may aid the clinician in rendering appropriate treatment.

We report a case of right retroperitoneal schwannoma diagnosed by guided FNAC in an elderly man who presented with right flank pain and slowly growing abdominal mass for a period of 40 years.

## Case Report

A 60-year-old male presented with history of vague abdominal pain since 6 years and mass per abdomen since 40 years. Pain was on and off and was felt around the umbilical and epigastric region, increasing after taking food, and was non-radiating. Mass was seen to occupy the right lumbar, right iliac fossa, epigastric and umbilical region with a firm consistency. The mass was regressing in size since 2 years. There was history of loss of weight and appetite since 4 months. He was a known alcoholic and smoker for 20 years.

Ultrasound scan showed a well-defined capsulated mixed echogenic retroperitoneal mass with areas of necrosis and calcification seen extending from the right paravertebral umbilical region. CT scan showed a large well-defined peripherally enhancing retroperitoneal hypodense mass with areas of peripheral calcifications noted in the right lower abdomen, causing mass effect suggestive of retroperitoneal schwannoma.

Ultrasound guided FNAC of the retroperitoneal mass was performed using a disposable 10-ml syringe and 22- gauge disposable lumbar puncture (LP) needle; at multiple sites in the mass, maintaining negative pressure. FNA smears were stained with hematoxylin and eosin (H and E), Papanicolaou stain (Pap) and May-Grünwald-Geimsa (MGG) stain.

### Cytology findings

**Figure 1:**
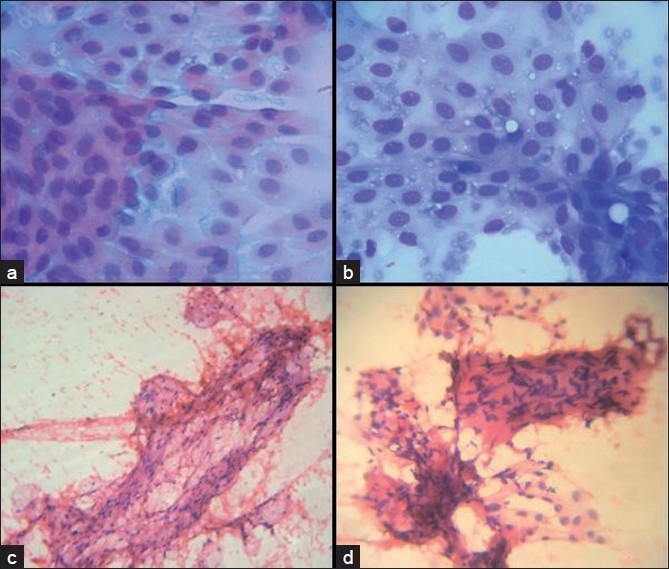
Microphotographs of FNA smears (a, b) polygonal cells with round nuclei and vacuolated cytoplasm (Pap and MGG, ×400), (c) cohesive clusters of spindle cells (H and E, ×40), (d) Verocay body and cyst macrophages (H and E, ×100)

Moderately cellular smear showed a few sheets of polygonal cells with round, uniform nuclei and vacuolated cytoplasm (cyst macrophages) [Figure 1a and b] along with fragments of fibroadipose tissue and occasional cohesive clusters of spindle cells with elongated nuclei [Figure 1c], Verocay body like arrangement of the spindle cells [Figure 1d], suggestive of benign spindle cell lesion, probably schwannoma.

The patient was taken up for surgery, and pre-operatively, the mass was identified in the right loin area, attached to right abdominal wall via mesentry. Ureter was identified medial to the mass. Posteriorly, mass was attached to the transverse process of the underlying vertebra, inferior venacava and to right common iliac artery. Debulking of the mass was done and sent for histopathology.

**Figure 2:**
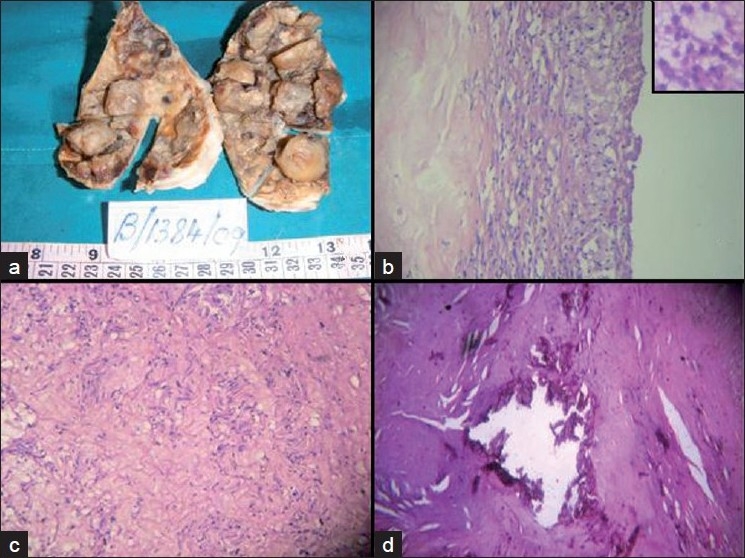
Gross photograph showing (a) two thick membranous masses with cut surface showing multiple nodules, (b) microphotograph showing thick fibrocollagenous wall with foamy histiocytes and hyalinization (H and E, ×100) (inset) cyst macrophages (H and E, ×400), (c) Antoni A areas with Verocay bodies (H and E, ×100), (d) foci of calcification (H and E, ×100)

Gross examination revealed two thick membranous soft tissue masses measuring 11×7×1.5 cm and 10×7×0.5 cm. External surface appeared smooth. Cut surface appeared ragged showing multiple nodules, the largest measuring 3×2.5×1 cm [Figure 2a]. Cut section of the nodule showed grey white to grey brown areas.

Histopathological examination revealed schwannoma surrounded by thick fibrocollagenous wall, showing collection of foamy histiocytes and areas of hyalinization [Figure 2b]. Verocay bodies (palisading nodules) with Antoni A (highly ordered cellular component, well -organised spindle cells in palisading pattern) [Figure 2c] and Antoni B (less cellular, loose textured pleomorphic cells against a myxoid background) were present focally. Degenerative changes like cystic degeneration, coagulative necrosis and foci of calcification [Figure 2d] were seen. The stroma showed infiltration by lymphoplasmacytic cells.

**Figure 3:**
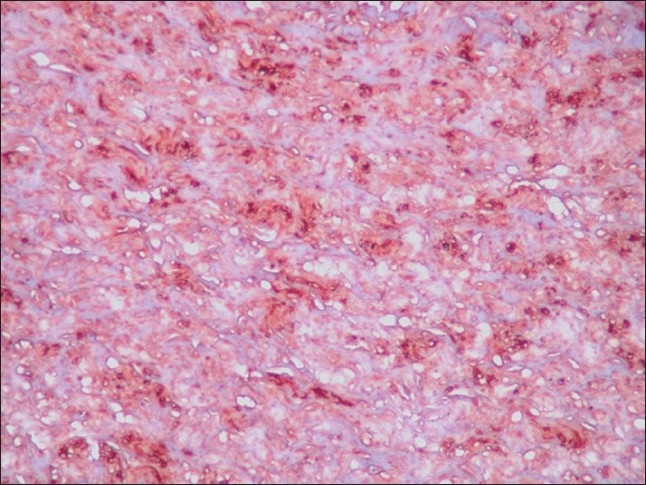
Photomicrograph showing S-100 positivity of Schwann cells (IHC, ×400)

Immunohistochemical staining for S-100 protein strongly highlighted the spindle cells [Figure 3]. There was no evidence of malignancy.

The final diagnosis was retroperitoneal schwannoma with cystic degeneration.

## Discussion

Schwannomas or neurilemmomas or neurinomas are benign, slow growing, solitary and well-encapsulated lesions that arise from the Schwann cells. Extracranial schwannomas present as solitary mass anywhere in the body. The common sites include the head and neck, the flexor surfaces of the upper and lower extremities, the posterior mediastinum in the thorax and on the trunk.[4] Cases involving the retroperitoneum, small bowel, extrahepatic biliary tree, pancreas, pelvis and sacrum have been reported as rare sites of occurrence.[5] Retroperitoneal schwannoma is a rare entity accounting for 0.3 - 3.2% of benign schwannomas.[6] Diagnosis of retroperitoneal schwannomas is difficult because they remain asymptomatic or present with late clinical manifestations. The pressure they exert on adjacent structures or nerves usually brings these tumors to clinical attention.[4]

Early symptoms of retroperitoneal tumors are vague and none can be considered diagnostic. The common symptoms include vague, poorly localised pain and discomfort. Atypical presentations are very rare and include flank pain, haematuria, headache, secondary hypertension and recurrent renal colic pain.[6,7] CT and MRI are widely used as imaging techniques in the evaluation of retroperitoneal soft tissue tumors.[4] CT scan typically shows well-defined low or mixed attenuation with cystic necrotic central areas.[7] MRI allows better visualisation of the tumor vascularity, but still these features may not be pathognomonic of schwannoma. So, preoperative diagnosis of retroperitoneal schwannoma is very difficult because of its site, asymptomatic clinical course, no specific diagnostic features on imaging studies.[3] Guided FNAC has emerged as an important diagnostic tool for appropriate diagnosis of retroperitoneal lesions. FNA cytological features of schwannoma have been well described and illustrated in literature. Classic features of benign lesion include fascicles of cohesive cells as well as a loose component, wavy or fibrillary, indistinct cytoplasm, and bipolar cells with cigar shaped or fishhook nuclei with blunt ends. The presence of nuclear palisading and Verocay bodies appears to be variable.[8,9] “Ancient” schwannoma, was initially mentioned by Ackerman and Taylor[10] as a degenerative change occurring in a long standing schwannoma, was characterised by nuclear hyperchromasia, mild nuclear pleomorphism, stromal oedema, fibrosis and xanthomatous changes leading to a misdiagnosis of malignancy in the aspirates.

Dahl *et al*[11] described “kern-loch” phenomenon or large intranuclear vacuoles, which appears to correlate with nuclear atypia in schwannoma. Mitoses have been described in association with “cellular” schwannoma, a variant of schwannoma, by Woodruff *et al*.[12] Grossly, schwannomas are usually solitary, well-circumscribed, firm, smooth surfaced tumors. As extracranial schwannomas are usually large, these tumors manifest with secondary degenerative changes due to the long duration resulting in waxing and waning of the tumor size as seen in the present case.[4,7]

Histologically, schwannomas may demonstrate biphasic pattern with compact areas of high cellularity (Antoni type A) and loose, hypocellular myxoid areas with microcystic spaces (Antoni type B). Cystic changes occur more commonly in retroperitoneal schwannomas (upto 66%) than in other retroperitoneal tumors. Other degenerative changes such as calcification, hemorrhage and hyalinization reported by other authors were also seen in our case.[7] Occasionally, the cyst wall may contain abnormal sinusoid and telangiectasia like vessels, endothelial proliferations with myxoid degeneration and necrosis.[13]

Heterogeneity caused by cystic degeneration designates the tumor as “ancient” schwannoma. Degeneration is due to central tumor necrosis as the schwannoma grows to a size beyond the capacity of its blood supply.[6] Calcification within the tumor mass, which has been reported in a single case literature, were seen in our case, and may not be considered as much of a diagnostic evidence. Immunohistochemically, these tumors show diffuse positivity for S100 protein in the cytoplasm of the tumor cells.[7] Malignant change in schwannomas is extremely rare, but when present, acts as high grade sarcomas producing local recurrence and distant metastasis. Malignant changes include mitosis, pleomorphism and blood vessel infiltration.[7] Malignant transformation (neurofibrosarcoma) is usually observed in cases with underlying Von Recklinghausen’s disease.[6] The differential diagnosis includes pancreatic cystic tumors, hepatic tumors, psoas abscess, liposarcomas, hemangiopericytomas.[4]

Wide surgical resection is advocated in cases of benign retroperitoneal schwannoma. Care must be taken in attempting removal of retroperitoneal and intrapelvic schwannomas.[6] There are a few reported cases in which the metastases occurred after resection of a histologically benign schwannoma.[14] Therefore, careful monitoring is recommended after removal of benign retroperitoneal schwannoma.
